# Supplementary material for: Red Blood Cell-Derived miR-93-5p Correlates with PD-1/PD-L1 Upregulation and Poor Prognosis in Lung Cancer
Source: medRxiv. 2025 Dec 15:2025.12.11.25342074. Preprint. [Version 1] doi: 10.64898/2025.12.11.25342074 (PMC12724151; doi:10.64898/2025.12.11.25342074)
Supplement: 1 [file NIHPP2025.12.11.25342074V1-supplement-1.pdf]

medRxiv preprint doi: https://doi.org/10.64898/2025.12.11.25342074; this version posted December 15, 2025. The copyright holder has placed this preprint (which was not certified by peer review) in the Public Domain. It is no longer restricted by copyright. Anyone can legally share, reuse, remix, or adapt this material for any purpose without crediting the original authors.

Supplementary File

Supplementary Table 1. Primary Antibodies Used for IHC

Targets	Host / Isotype	Company
$CD8^{+}$ T cells	Mouse / IgG	Thermo Fisher Scientific
PD-1	Rabbit / IgG	Thermo Fisher Scientific
PD-L1	Rabbit / IgG	Thermo Fisher Scientific
CD69	Mouse / IgG	Thermo Fisher Scientific
IFN-γ	Rabbit / IgG	Thermo Fisher Scientific
TNF-α	Mouse / IgM	Abcam

Supplementary Table 2. Pearson's Correlation Coefficients between miR-93-5p and Immune Factors in Lung Cancer Patients

miR-93-5p	PD-L1	PD-1	CD8	CD69	IFN-γ	TNF-α
In RBCs	r = +0.482*	r = +0.421*	r = −0.294	r = −0.261*	r = −0.412*	r = −0.367*
In RBC-derived exosomes	r = +0.458*	r = +0.396*	r = −0.275	r = −0.239*	r = −0.398*	r = −0.346*
In tumor tissues	r = +0.312*	r = +0.287*	r = −0.203	r = −0.228*	r = −0.276*	r = −0.241*

*Statistically significant correlations ($p < 0.05$).

Supplementary Table 3. Multivariate Cox Proportional Hazards Regression Analysis of miR-93-5p Expression and Immune Factors, and Stags

Variable	Hazard Ratio	95% Confidence Interval	p-value
miR-93-5p in RBCs	1.923	1.218 - 3.038	0.016
miR-93-5p in RBC-derived exosomes	1.784	1.153 - 2.911	0.021
miR-93-5p in tumor tissues	2.103	1.327 - 3.498	0.011
PD-L1	1.847	1.152 - 3.003	0.018
PD-1	1.648	1.104 - 2.503	0.025
CD69	1.123	0.972 - 1.298	0.063
IFN-γ	0.943	0.893 - 0.994	0.082
TNF-α	0.911	0.872 - 0.963	0.073
Age (years)	1.023	1.002 - 1.041	0.083
Gender (Male vs. Female)	1.104	0.748 - 1.602	0.653
Disease Stage (III-IV vs. I-II)	3.002	1.802 - 5.003	0.037

Hazard ratios were estimated using multivariate Cox regression.

medRxiv preprint doi: https://doi.org/10.64898/2025.12.11.25342074; this version posted December 15, 2025. The copyright holder has placed this preprint (which was not certified by peer review) in the Public Domain. It is no longer restricted by copyright. Anyone can legally share, reuse, remix, or adapt this material for any purpose without crediting the original authors.

Supplementary Table 4. Associations of miR-93-5p Expression Levels with Immune Factors, Clinical Characteristics, and OS

**Factors**	**miR-93-5p in RBCs (p-value)**	**miR-93-5p in RBC-derived exosomes (p-value)**	**miR-93-5p in tumor tissues (p-value)**
PD-1	0.012	0.013	0.011
PD-L1	0.011	0.012	0.01
$CD8^+$	0.081	0.092	0.076
CD69	0.003	0.002	0.002
IFN-γ	0.002	0.003	0.002
TNF-α	0.004	0.003	0.004
Disease Stage	0.024	0.018	0.016
Overall Survival (OS)	0.015	0.011	0.009
Age (Mean ± SD, years)	0.648	0.652	0.645
Gender	0.741	0.736	0.734
Histological Type	0.284	0.391	0.308

Continuous variables (e.g., age, immune factors) were analyzed using Pearson's correlation. Categorical variables (e.g., gender, disease stage, histological type, survival outcomes) were compared using the Chi-square test. Overall survival (OS) associations were assessed using the log-rank test and Cox proportional hazards regression. $p < 0.05$ was considered statistically significant.

Supplementary table 5. Multivariate Cox Proportional Hazards Regression Analysis of Combined miR-93-5p expression across multiple compartments on Overall Survival (OS)

**Variables**	**Hazard Ratio (HR)**	**95% Confidence Interval (CI)**	**p-value**
miR-93-5p in RBCs	1.923	1.218 - 3.038	0.016
miR-93-5p in RBC-derived exosomes	1.784	1.153 - 2.911	0.021
miR-93-5p in tumor tissues	2.103	1.327 - 3.498	0.011
Combined miR-93-5p expressions	2.873	1.427 - 4.639	0.015
